# Physical implementation of oblivious transfer using optical correlated randomness

**DOI:** 10.1038/s41598-017-08229-x

**Published:** 2017-08-16

**Authors:** Tomohiro Ito, Hayato Koizumi, Nobumitsu Suzuki, Izumi Kakesu, Kento Iwakawa, Atsushi Uchida, Takeshi Koshiba, Jun Muramatsu, Kazuyuki Yoshimura, Masanobu Inubushi, Peter Davis

**Affiliations:** 1 Department of Information and Computer Sciences, Saitama University, 255 Shimo-okubo,Sakura-ku, Saitama City, Saitama, 338-8570 Japan; 2 NTT Communication Science Laboratories, NTT Corporation, 3-1 Morinosato, Wakamiya, Atsugi-Shi, Kanagawa, 243-0198 Japan; 3 Department of Information and Electronics, Graduate school of Engineering, Tottori University 4-101 Koyama-Minami, Tottori, 680-8552 Japan; 4 Telecognix Corporation, Japan, 58-13 Shimooji-cho, Yoshida, Sakyo-ku, Kyoto, 606-8314 Japan

## Abstract

We demonstrate physical implementation of information-theoretic secure oblivious transfer based on bounded observability using optical correlated randomness in semiconductor lasers driven by common random light broadcast over optical fibers. We demonstrate that the scheme can achieve one-out-of-two oblivious transfer with effective key generation rate of 110 kb/s. The results show that this scheme is a promising approach to achieve information-theoretic secure oblivious transfer over long distances for future applications of secure computation such as privacy-preserving database mining, auctions and electronic-voting.

## Introduction

With the rapid evolution of big data and cloud computing systems there is increasing interest in practical schemes for secure operations on information on large scales. One example is secure computation which would allow computation of functions over data without revealing the data^1–15^. Practical large scale implementations of secure computation are needed to realize applications such as private information retrieval, privacy preserving database mining, auctions, and electronic voting systems.

A key component for secure computation is oblivious transfer. Oblivious transfer is message transfer in which a sender sends encoded messages in such a way that the receiver can only decode some of the messages and the sender does not know which messages were decoded. The original notion of oblivious transfer using an erasure channel was given by Rabin^16^. Later, one-out-of-two oblivious transfer was considered by Even *et al*.^17^. Naor and Pinkas^18, 19^ gave an oblivious transfer protocol based on the Diffie-Hellman assumption, where the protocol relies on computational complexity. It has been known that the Naor-Pinkas protocol is time-consuming, and large amount of computation is required. The oblivious transfer extension technique of Ishai *et al*.^20^ and follow up work has been aimed at achieving faster and more efficient oblivious transfer.

Various schemes for oblivious transfer based on information-theoretic security have also been proposed. Information-theoretic oblivious transfer can be secure with respect to adversaries that are computationally unbounded. Moreover, information-theoretic oblivious transfer can be future proof in the sense that secrets will not be revealed by future advances in computational power. Information-theoretic schemes are based on the idea of distilling a secret bit, or string of secret bits, from a statistical advantage in correlation of bits acquired from a probabilistic system. Different models can be distinguished based on specific features of the probabilistic model of the system. Following the original notion of the erasure channel^16^, there have been schemes proposed based on noisy channels^21–24^, bounded storage^25^, wireless communication systems^26^, quantum mechanical systems^27–30^, and network behaviors^31, 32^. In the noisy channel model, users observe a random sequence from a common source (such as a broadcast satellite), but the detected bits are different with a certain probability due to noise in the channel. In this case, sets of matching bits can be identified (“distilled”) by techniques such as comparing results of hash functions. The bounded storage model assumes that users observe the same random sequence but some observations are randomly dropped due to storage limits. Matching bits can be identified by exchanging sequence labels of stored bits. Quantum schemes for oblivious transfer are based on a random choice of observation function in a quantum mechanical system. Matching bits can be identified by transmitting observation parameter information together with observation sequence labels.

However, feasible schemes for physical implementation of information-theoretic oblivious transfer over long distance with high bit rate, which is necessary for practical use, are still lacking. Information-theoretic schemes are difficult to implement because of the difficulty of generating large volumes of entropy in a way that corresponds to a reliable probabilistic model such as an erasure channel^16^ or a binary symmetric channel^21^ and its variant^22^. Entropy sources based on analog physical signals depend on the bandwidth and dynamic ranges of analog-to-digital detectors, as can be seen for example in the ongoing efforts to build non-deterministic random number generators operating above Gigabit per second (Gbps)^33–35^. Implementation of channel models such as Gaussian or wireless channels^23–25^ rely on assumptions about the stochastic state of the channel which are difficult to guarantee in practice, especially in open environments.

Recently, it has been shown that a correlated random source suitable for information-theoretic security applications can be implemented using random light transmitted large distances over optical fiber and injected into semiconductor lasers^36, 37^. The parameter-dependent synchronization properties of complex dynamical systems driven by a common random signal^38–41^ are used to realize a correlated random source for secure key distribution based on information-theoretic security^42^. The scheme assumes a random choice of observation function in a classical optical system where the same choices result in detecting identical bits, and different choices result in uncorrelated bits. Matching bits can be identified by communicating information about observation choices over an open communication channel. The correlated randomness required for information-theoretic security relies on the property of bounded observability^43–45^, whereby the number of simultaneous observations of a system by a single user is limited and the state of the observed system cannot be known by any other means. These properties can be feasible due to technological limits of observing a physical system. The proposal in refs 36 and 37 was based on the technological difficulty of completely observing broadband random light. The physical system was implemented using common broadband random light transmitted over optical fiber. Users observe the received light by injecting it into a laser device and detect the output to obtain binary bits. Random choice of observation function was implemented with a random choice of a laser parameter, so that identical choices result in detecting identical bits, and different choices result in uncorrelated bits. Secure key distribution using this scheme has been demonstrated experimentally for nodes separated by over 120-km of optical fiber at a key generation rate of 64 kb/s^37^.

The bounded observability scheme is similar to a quantum scheme in the sense of generating a sequence of parameter-bit pairs. However, the difficulty of transmitting quantum states over large distances currently limits the separation distance to tens of kilometers. The optical implementation is feasible for generating correlated randomness over hundreds of kilometers using conventional optical amplifiers, since the scheme uses a classical optical state rather than a quantum optical state.

This physical implementation of a source of correlated randomness in an optical fiber system was a breakthrough in both speed and reliability over large distances, bringing information-theoretic schemes closer to the regime of practical feasibility. The work in this paper builds on this breakthrough and shows how to harness it for information-theoretic oblivious transfer. Specifically, we present the first experimental demonstration of information-theoretic oblivious transfer using an optical correlated random source based on the bounded observability model.

## Correlated random optical source

**Figure 1:**
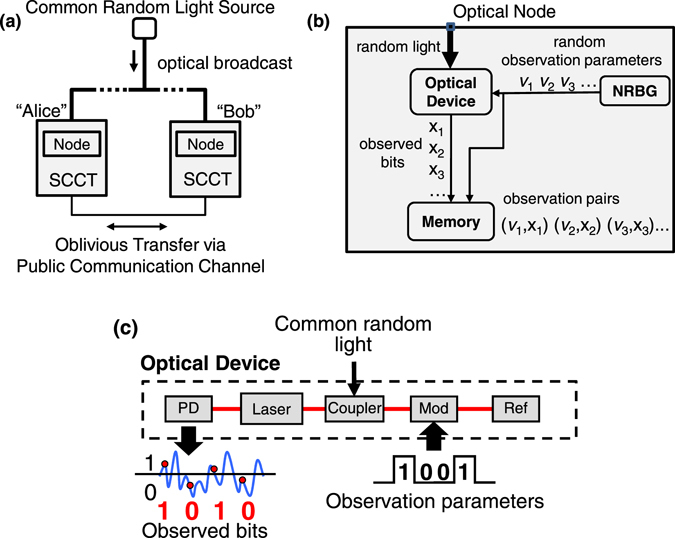
Schematic diagram of system for oblivious transfer using optical correlated randomness. (**a**) Total system, (**b**) optical node, and (**c**) optical device. (**a**) SCCT: secure communication and computation terminal. (**b**) NRBG: non-deterministic random bit generator. (**c**) Mod: optical phase modulator, PD: photodetector, and Ref: optical reflector.

Figure 1 shows the proposed scheme for oblivious transfer using optical correlated randomness. A common random signal can be broadcast through optical fibers or free space (Fig. 1(a)). Two legitimate users Alice and Bob each have optical nodes (Fig. 1(b)) consisting of an optical device (Fig. 1(c)) driven by the common random optical signal. Alice and Bob independently acquire and record many observations at their respective optical nodes, where each observation consists of the values of the random observation parameters and the corresponding observed bits (Fig. 1(b)). The bits are highly correlated if the observation parameters are identical and uncorrelated if the observation parameters do not match. After recording their observations, Alice and Bob execute an oblivious transfer protocol over an open channel, and use oblivious transfer to execute secure computation. Note that the common random broadcast signal can be provided by either Alice or a trusted third party, but not by Bob.

First we describe the scheme for acquiring random sequences from the laser optical system. Alice and Bob each have a semiconductor laser with an external cavity containing a phase modulator (Fig. 1(c)). Each laser has a variable parameter *v* (e.g., optical-feedback phase) with different values (e.g., 0 and 1, corresponding to zero- and π-phase shift, respectively). A random broadband light *S* is broadcast to the users. They each inject the received light *S* into their laser. Each laser generates an optical output, which depends on both *S* and *v* and has the following property of correlated randomness: the temporal waveforms of the laser outputs are strongly correlated if the parameter values of Alice and Bob are the same (*v*_*A*_ = *v*_*B*_), and mutually uncorrelated if the parameter values are different (*v*_*A*_ ≠ *v*_*B*_). Alice and Bob independently select their parameter values *v*_*A*_ and *v*_*B*_ at random. Alice and Bob simultaneously sample and quantize their laser outputs to extract bits *x*_*A*_ and *x*_*B*_ (0 or 1), respectively. Alice and Bob then store the parameter-bit pairs (*v*_*A*_, *x*_*A*_) and (*v*_*B*_, *x*_*B*_) in their data recorders (Fig. 1(b)). They repeat this procedure many times, injecting the continuously varying nonrepeating random light *S* to their lasers with parameters randomly selected each time *t*, to acquire sequences of the parameter-bit pairs (*v*_*A*_(*t*), *x*_*A*_(*t*)) and (*v*_*B*_(*t*), *x*_*B*_(*t*)), *t* = 1, 2,…, *m*, respectively. Due to the synchronization properties of the two lasers, the same bits are obtained (*x*_*A*_(*t*) = *x*_*B*_(*t*)) between Alice and Bob when the parameters are the same (*v*_*A*_(*t*) = *v*_*B*_(*t*)), but when the parameters are different (*v*_*A*_(*t*) ≠ *v*_*B*_(*t*)), the probability of the bits being equal is just 0.5.

The security of the oblivious transfer scheme with regard to malicious attacks relies on two physical limitations on observations in the optical system^36, 37^. (i) No one can continuously measure and record the entire common random light in order to repeat the observations of Alice or Bob *after* the parameter settings have been exchanged. (ii) No one can simultaneously observe the outputs for all possible parameter values while the common random light is being broadcast. The limitation (i) can be achieved by delaying the parameter exchange long enough to make it impossible to store the entire length of common random signal in an optical delay line or ring buffer, and by using broadband random light with a fluctuation bandwidth which is too broad to accurately and continuously electronically observe and record its fast temporal variation with current technology. The second limitation (ii) can be achieved by increasing the number of possible parameter values of the receiver optical device, to make it practically infeasible to prepare the number of devices required to simultaneously observe all possible cases. An example of a scalable receiver device based on cascaded laser systems is described in the supplementary material.

We note that in order to realize information-theoretic security, it is not necessary to prevent the attacker from obtaining some information, or to know which information they obtain. It is only necessary to estimate the amount of information that they might obtain, and apply a suitable privacy amplification procedure. There is a fundamental trade-off between the amount of partial information leak that is allowed and the rate at which secure keys can be generated for oblivious transfer.

### One-out-of-two oblivious transfer protocol

One-out-of-two oblivious transfer means the following conditions hold for legitimate users Alice and Bob communicating through an authenticated public channel. (i) Alice sends two encoded messages to Bob, and Bob can only decode one of them, but not the other one. (ii) Alice cannot know which message Bob has decoded.

**Figure 2:**
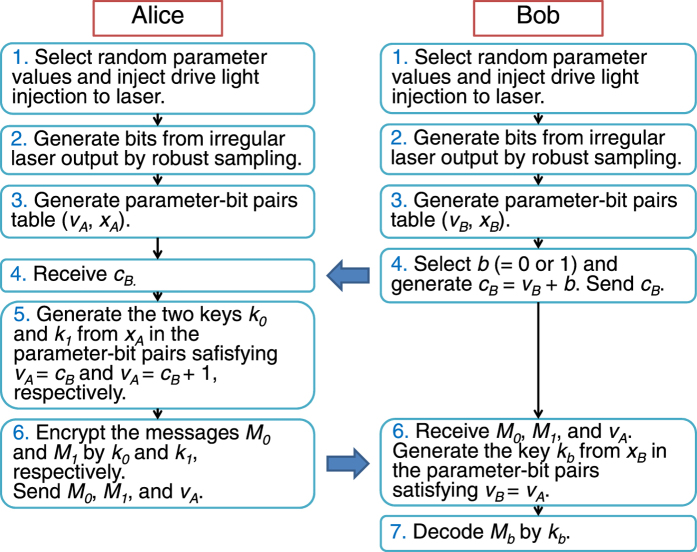
Protocol of one-out-of-two oblivious transfer. +Denotes bitwise exclusive-OR operation. ν_*Α,Β*_: random observation parameters for Alice and Bob. *x*_*A,B*_: observed bits for Alice and Bob.

We propose the following protocol for one-out-of-two oblivious transfer (Fig. 2). Consider Alice has two messages, *M*_0_ and *M*_1_, to send to Bob, and Bob decides a binary value *b* = 0 or 1 according to which message *M*_*b*_ he intends to decode. First, Bob sends the following parameter set {*c*_*B*_(*t*)}^*m*^_*t* =1_ to Alice,

1 $${c}_{B}(t)={v}_{B}(t)+b$$

where + denotes bitwise exclusive-OR (XOR) operation. That is, Bob sends his parameter set {*c*_*B*_(*t*)}^*m*^_*t* =1_ = {*v*_*B*_(*t*)}^*m*^_*t* =1_ as it is to Alice if he intends to obtain *M*_0_, or sends the inverted values {*c*_*B*_(*t*)}^*m*^_*t* =1_ = {*v*_*B*_(*t*) + 1}^*m*^_*t* =1_ if he intends to obtain *M*_1_. After Alice receives Bob’s parameter set {*c*_*B*_(*t*)}^*m*^_*t* =1_, Alice creates two cryptographic keys *k*_0_ and *k*_1_, using observed bits *x*_*A*_(*t*) corresponding to the parameter matches *v*_*A*_(*t*) = *c*_*B*_(*t*) and *v*_*A*_(*t*) = *c*_*B*_(*t*) + 1 respectively. That is, for the key *k*_0_, Alice uses the bits *x*_*A*_(*t*) observed at times *t* when *v*_*A*_(*t*) = *c*_*B*_(*t*), and for the key *k*_1_, Alice uses the bits *x*_*A*_(*t*) observed at times *t* when *v*_*A*_(*t*) = *c*_*B*_(*t*) + 1. Then Alice encrypts the message *M*_0_ with the key *k*_0_ and the message *M*_1_ with the key *k*_1_, and sends the two encrypted messages and her original parameter set {*v*_*A*_(*t*)}^*m*^_*t* =1_ to Bob. Finally, Bob creates a key using the bits *x*_*B*_(*t*) for the sampling time *t* such that *v*_*B*_(*t*) = *v*_*A*_(*t*). Bob’s key will be identical to Alice’s key *k*_*b*_, where *b* = 0 or 1 is the value Bob initially decided. Thus Bob is able to decode the message *M*_*b*_ that he intended but not the other message, and Alice does not know which of the two messages Bob can decode.

This protocol can be extended to include privacy amplification to make it robust against statistical bias in bits and secure against malicious attacks that might learn partial information about the observations of other users. Details are given in the Methods section.

## Results

### Experimental implementation of oblivious transfer

The components of the optical system are feasible for practical optical fiber communication (See Methods for details). The lasers are semiconductor lasers operating at wavelengths used for long range optical communication, 1.5 microns. The common random optical signal is generated using a laser with phase modulated by a random noise generator^38^, and sent to Alice and Bob over optical fiber. In the optical nodes of Alice and Bob, the received common random signal is injected into a laser with an external cavity under conditions for so-called generalized synchronization^35, 39^, such that after a transitory evolution the fluctuating dynamical state of the laser is determined by the combination of drive signal and the optical feedback phase^38, 46^. The optical feedback phase can be controlled by an external electrical signal and is used as the observation parameter for the oblivious transfer protocol.

**Figure 3:**
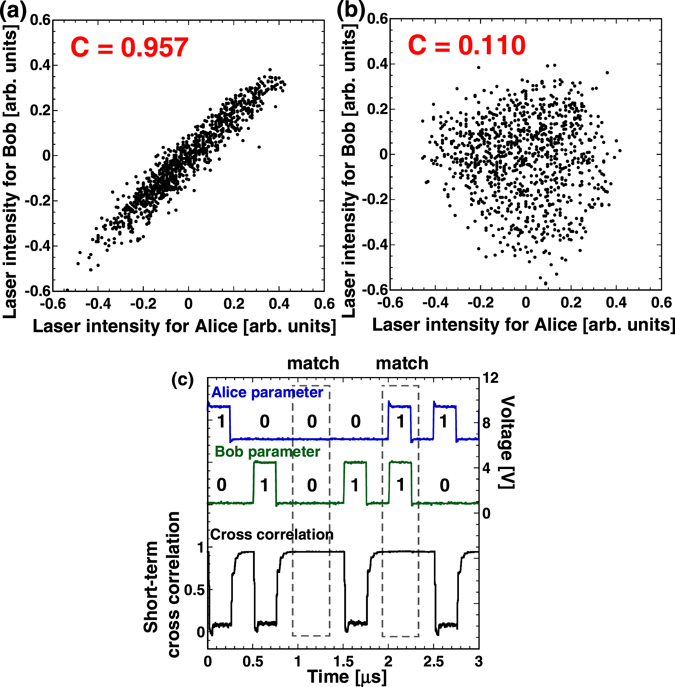
Experimental result of optical correlated randomness. (**a**,**b**) Correlation plots of temporal waveforms of Alice’s and Bob’s laser outputs. *C* indicates cross-correlation value. (**a**) Same parameter selection, and (**b**) different parameter selection. (**c**) Example of parameter switching. Alice’s and Bob’s parameter values and the short-term cross-correlation between Alice’s and Bob’s temporal waveforms are shown.

Figure 3(a) and (b) show examples of correlation plots between temporal waveforms of the output from the lasers of Alice and Bob. The temporal waveforms are correlated when the parameters of Alice and Bob are matched, and uncorrelated when the parameters are mismatched. Figure 3(c) shows the change of short-term cross correlation of the temporal waveforms between the two laser outputs for Alice and Bob when the parameters are repeatedly shifted (0 and 1 correspond to zero and π phase shift, respectively) in independent random sequences. The feedback phase of the lasers is modulated with a return-to-zero (RZ) format at a frequency of 2 MHz to ensure the transient time of synchronization. After a short transient time of each switch, the correlation settles to a steady value. High values of the short-term cross correlation are obtained when the phase parameters match, and low correlation values are observed when the parameters do not match. The intensity of the fluctuating laser output is sampled with a 1-bit digitizer at a fixed time after the parameter switch longer than the correlation relaxation time. In order to reduce the effect of noise and synchronization errors, we implemented a method known as robust sampling for the 1-bit sampler^36, 37^. This method uses two intensity thresholds - a value above the upper threshold is detected as 1, a value below the lower threshold is detected as 0, and the sample is discarded otherwise. Repeated switching and sampling at the rate of 2 MHz result in fast generation of a sequence of parameter-bit pairs including timestamps that are stored in the user’s memory.

Next we examine the oblivious transfer using the parameter-bit pairs. Alice sends two messages *M*_0_ and *M*_1_ that are random bits encrypted with the keys *k*_0_ and *k*_1_, respectively. We assume that Bob selects *b* = 1 and generates key *k*_1_ to decode the message *M*_1_. Table 1 shows a typical result of the oblivious transfer in the case where Alice and Bob generate a 3,600-bit key, and Bob recovers the message *M*_1_ correctly without errors. In this case, we also evaluate the randomness of the generated binary key *k*_1_. The ratio of ‘0’ bits in *k*_1_ is 0.4933, which satisfies the criterion of the bit bias for random bits, 0.5 ± 3/(2 $\sqrt{N}$), where *N* is the number of bits^47^.

**Table 1:** Result of oblivious transfer at the bit generation rate of 110 kb/s. Left column: legitimate users (comparison between Alice’s key *k*_*1*_ and Bob’s key *k*_*1*_).

	Legitimate users	Bob’s attack 1	Bob’s attack 2
	Alice *k*_*1*_, Bob *k*_*1*_	Alice *k*_*0*_, Bob *k*_*1*_	Alice *k*_*0*_, Bob *k*_*2*_
Number of generated bits	3600	3600	3600
Bit error rate	0	0.491	0.386
Ratio of 0	0.4933	0.4933	0.3883
Mutual information between Alice and Bob	1	0.0002	0.0269

Middle column: Bob’s attack 1 (comparison between Alice’s *k*_*0*_ and Bob’s *k*_*1*_). Right column: Bob’s attack 2 (comparison between Alice’s *k*_*0*_ and Bob’s *k*_*2*_). Bob generates *k*_*2*_ using the bits that he observed at the timestamps *t* of observations used by Alice for *k*_*0*_.

We consider two possible attacks by Bob to decode the other message *M*_0_, under the assumption of a semi-honest user: (i) Bob uses his key *k*_1_ to estimate Alice’s key *k*_0_, (ii) Bob generates a new key (called *k*_2_) using the bits that he observed at the timestamps *t* of observations used by Alice for *k*_0_. (From *v*_*A*_ and *v*_*B*_ Bob knows the timestamps *t* of observations used by Alice for *k*_0_ as well as *k*_1_). These results are also shown in Table 1. For both of the attacks, the bit error rates (BERs) between Alice’s *k*_0_ and Bob’s *k*_1_ (or *k*_2_) are close to 0.5, indicating that Bob cannot decode the message *M*_0_. It is found that the second attack is more effective than the first one, since BER is slightly lower (BER = 0.386). The mutual information between Alice’s *k*_0_ and Bob’s *k*_1_ and *k*_2_ generated in the two attacks are 0.0002 and 0.0269, respectively. Similarly, the second attack is more effective since there is a small correlation (see Fig. 3(b)) between the temporal waveforms used for generating the keys *k*_0_ and *k*_2_. The small information leakage in these attacks can be reduced by privacy amplification^48, 49^ (See the Methods section for details).

**Figure 4:**
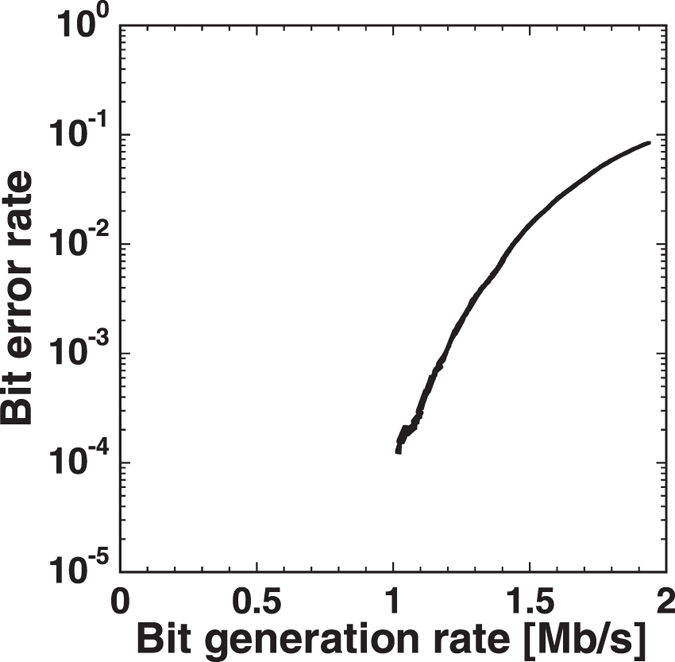
Relation between bit error rate (BER) and bit generation rate (BGR) for parameter-bit observation pairs of optical correlated random source for oblivious transfer. The two threshold values of the robust sampling are changed.

Next, we evaluate the key error ratio, the ratio of cases where Alice’s key *k*_1_ and Bob’s key *k*_1_ do not match due to bit errors in the optical samples. The proportion of bit errors of the optical samples depends on the two threshold values of the robust sampling - larger threshold separation results in lower BER and lower observation-pair (bit) generation rate^36, 37^. Measured values of BER and bit generation rate (BGR) are shown in Fig. 4. With 2 MHz sampling clock and robust sampling with BGR of 1 Mb/s or less, the BER is less than 10^−4^. The results in Table 1 were obtained with BGR of 220 kb/s, for which the BER is much less than 10^−4^. Considering a match probability of 0.5, and a negligible bit error, the maximum key generation rate can be estimated as 110 kb/s.

### Secure computation

Next we demonstrate the use of oblivious transfer to implement two-party secure computation, using the one-out-of-two oblivious transfer and Yao’s garbled circuit^1, 2, 7^. Technical details are provided in the Methods section and the supplementary material. Our purpose here is to experimentally demonstrate one way of operating the oblivious transfer system in the context of secure computation, and to evaluate the latency of the oblivious transfer system in this context.

The secure computation implemented is a comparison of two numbers, (This is known as the millionaire problem^1, 2^, as it corresponds to Alice and Bob comparing two numbers representing their respective wealth, to determine which is larger, without revealing their numbers to each other). The multi-bit digital comparison is translated into an encrypted Boolean circuit, called a “garbled circuit”, which can be evaluated without the evaluator knowing all the inputs by making use of oblivious transfer. In the garbled circuit protocol, one of the users (Alice) encrypts each logical gate operation as an encrypted “garbled” logical table with logical values of input wires replaced by random bit strings (called labels), and sends the garbled circuit to the other user (Bob) together with the random labels corresponding to her input bits. Using one-out-of-two oblivious transfer for each input bit, Bob is able to get from Alice the label corresponding to the value of his input bit without learning the other label and without Alice knowing what his bit is. Then Bob is able to evaluate the garbled circuit to obtain the labels of the circuit output wires, which can be decoded by Alice or Bob.

**Figure 5:**
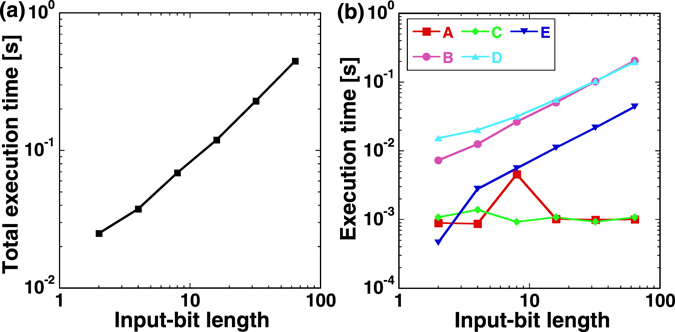
(**a**) Total execution time of secure comparison with garbled circuit for different input-bit lengths of 2-bit to 64-bit. (**b**) Execution time of each procedure for secure comparison. A (red squares): construction of logic circuit, B (pink circles): construction of garbled circuit, C (green diamonds): construction of Alice’s garbled input, D (light blue triangles): construction of Bob’s garbled input via oblivious transfer, and E (dark blue inverted triangles): execution of garbled circuit.

We simulate the garbled circuit protocol on a personal computer and evaluated the scheme together with the implementation of oblivious transfer. Figure 5(a) shows the total execution time for input-bit lengths of 2-bit to 64-bit. We found that the total execution time is proportional to the input-bit length on the log-log scale. We achieve 32-bit and 64-bit secure comparison in 0.23 and 0.45 seconds, respectively. Figure 5(b) shows the execution time for each procedure in the secure computation. The construction of the garbled circuit (B in Fig. 5(b)) and execution of the oblivious transfer (D in Fig. 5(b)) are the most time-consuming procedures. The execution time of the oblivious transfer is proportional to the input-bit length for large input-bit lengths. The time required for the oblivious transfer procedure is 0.2 seconds for 64-bit secure comparison, and corresponds to 44% of the total execution time (0.45 seconds). The time for the oblivious transfer was measured under the condition where the observation parameter-bit pairs are already stored in the computer. The pairs were generated in advance with the same sampling conditions as for Table 1. Hence, the time to generate the observation parameter-bit pairs required for each 100-bit oblivious transfer can be estimated as 100 bits-per-key/110 kb/s = 0.00091 sec, and the time to generate the observation parameter-bit pairs required for oblivious transfer of 64 input labels for 64-bit computation can be estimated as 0.058 sec, which is smaller than the oblivious transfer procedure time (0.2 sec).

## Discussion

Here we discuss a number of features of the implementation of oblivious transfer and its possible future extensions.

The raw parameter-bit-pair generation rate has an upper limit depending on the parameter switching rate. The parameter switching rate is limited by the transient time of synchronization of the laser outputs after the parameter values are switched. For the lasers used in the experiment, the transient synchronization time is several tens of nanoseconds. The transient time could be reduced by using lasers with shorter external cavity length such as photonic integrated devices^50, 51^.

The raw parameter-bit-pair generation rate is comparable with a recent information-theoretic oblivious transfer system based on a quantum scheme^30^. In addition, this oblivious transfer scheme has an advantage of the capability of long optical fiber transmission over hundreds of kilometers, much longer than quantum-based oblivious transfer^30^. It has been reported that no significant degradation of the statistical properties of the optical correlated randomness due to the propagation and amplification operations was observed in an experiment over 120 km of optical fiber with optical amplifiers^36, 37^. This fact indicates that the scheme of optical correlated randomness is feasible for stable operation of oblivious transfer over long distances in large-scale optical fiber networks, which is a practical important advantage over other techniques.

We consider the effect of robust sampling and bit error on the oblivious transfer rate. In the experiment errors were not detected due to the use of robust sampling. If we allow for a message to be resent with new keys when the oblivious transfer fails due to bit error, then it may be feasible to use a less strict robust sampling condition with a higher BER to achieve a higher BGR. The probability of key success for a key of bit-length *L*, can be estimated as (1-BER)^*L*^, and the effective key rate can be estimated as BGR × 0.5 × (1-BER)^*L*^, where 0.5 is parameter-match rate. For example, if the key length is 100 bits, the BGR is 1 Mb/s, and BER is 10^−4^, then the effective key rate is 495 kb/s, which is significantly larger than the value of 110 kb/s reported above for the case analyzed in Table 1.

Next, we consider the effective of bit bias. In the experiment statistically-significant bit bias was not observed. If there was a significant bias, the technique of privacy amplification^48, 49^ could be used to generate information-theoretic secure keys with arbitrarily small bit bias. In the Methods section we explain a method of privacy amplification for oblivious transfer and also provide an estimate of the corresponding key generation rate.

Privacy amplification is also essential for achieving security against physical attacks by malicious users that have physical (technological) limits. The experiment in this paper corresponds to the basic case where we assume that Alice and Bob are semi-honest, in the sense that they obey the protocol and do not act maliciously by using other physical measurements to obtain more information about the system that would allow them to know the other user’s bits after the parameter exchange. As mentioned earlier, by using privacy amplification, the oblivious transfer can be made information-theoretic secure against a malicious attacker that obtains partial information. It is not necessary to prevent the attacker from obtaining some information or to know which information they obtain. It is only necessary to estimate an upper bound on the amount of information they can obtain.

We consider two types of physical attacks by malicious users. First, an attacker may make multiple simultaneous observations, using multiple devices with different parameter settings (known as “multiple observation attack”^45^ or “sampling attack”^43^). To prevent this attack, the number of possible parameter settings needs to be increased so that it exceeds the upper estimate of the number of receiver devices that the malicious user can operate simultaneously. In fact, it is effective to increase the number of the laser stages in the cascaded laser system, because the malicious user needs to increase exponentially the number of his/her cascaded laser systems to perform the perfect multiple observation attack^37^ (See the supplementary material for details).

For the second attack, an attacker may reproduce the drive signal after the parameter exchange (known as “replay attack”). To prevent this attack, the bandwidth of the drive signal needs to be increased so that it exceeds the upper estimate of the length of signal that can be recorded by the user for replay after the parameter exchange. In the case of an attack using optical memory, the users can prevent this attack by delaying the parameter exchange longer than the propagation time or decay time of the signal in the optical memory. In the case of heterodyne detection, the attacker would need to record the modulation of the optical drive signal at least within the principal frequency band of the response laser^52, 53^. A successful attack by a malicious user against the laser used in this experiment would require an extremely advanced digitizer and data streaming system able to capture at least 10 GHz of modulation bandwidth^54^. In addition, a method has been proposed to increase the principal frequency band up to much broader bandwidths^52^ and this is a challenging area for future theoretical and experimental innovation in laser dynamics.

Finally, we note that as our example implementation of secure computation, we used the Yao’s garbled circuit which is based on computational security, rather than information-theoretic security. We note that the fundamental security assumptions of information-theoretic oblivious transfer and computational garbled circuit are different, and a complete implementation of information-theoretic secure computation is an important goal for the future. The scheme proposed by Kolesnikov *et al*. significantly reduces the computational cost of information-theoretic secure computation^55^, so this scheme could be feasible if the circuit size is not too large. We consider that this method would be a good candidate for future complete implementation of information-theoretic secure two-party computation.

## Conclusion

We demonstrate physical implementation of information-theoretic secure oblivious transfer based on bounded observability with optical correlated randomness. The implementation is realized using semiconductor lasers driven by common random signals broadcast over optical fiber. We experimentally demonstrated oblivious transfer at the effective key generation rate of 110 kb/s for the fundamental case of semi-honest users with a single binary-valued observation parameter and discussed the potential for realizing security against physical observation attacks by malicious users. This scheme is a promising approach to achieve information-theoretic secure oblivious transfer over long distances for future applications of secure computation such as privacy-preserving database mining, auctions and electronic-voting.

## Methods

### Experimental setup for oblivious transfer

**Figure 6:**
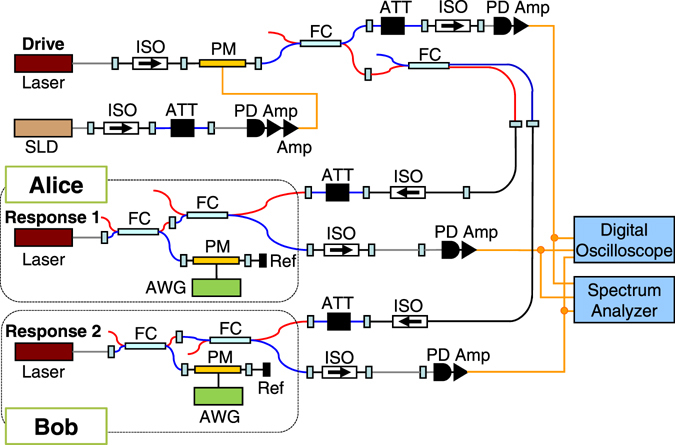
Experimental implementation of optical correlated random system based on optical fiber components for oblivious transfer. Amp: electronic amplifier, ATT: optical attenuator, AWG: arbitrary waveform generator, FC: fiber coupler, ISO: optical isolator, PD: photodetector, PM: phase modulator, Ref: fiber mirror reflector, SLD: super-luminescent diode.

The experimental setup of the optical correlated random system based on optical fiber components for oblivious transfer is shown in Fig. 6. We use three semiconductor lasers. The lasers are single-mode distributed-feedback (DFB) lasers (NTT Electronics, NLK1C5GAAA, the optical wavelength of 1547 nm) with external optical injection and optical feedback^36, 37^. One laser is used for a common drive signal (called Drive laser) and the other lasers are used for Response lasers. Each legitimate user (Alice or Bob) has a Response laser (called Response 1 and 2 lasers). The injection currents are set to 30.00 mA (2.84 *I*_*th*_), 12.30 mA (1.31 *I*_*th*_), and 12.68 mA (1.34 *I*_*th*_) for the Drive, Response 1, and 2 lasers, respectively, so that the relaxation oscillation frequencies of the Response lasers are as similar as possible, where *I*_*th*_ is the injection current at the lasing threshold. The relaxation oscillation frequencies are 5.8, 1.8, and, 1.8 GHz for the Drive, Response 1, and 2 lasers, respectively.

The Response 1 and 2 lasers are subject to a common random drive signal. We use a phase modulator (PM), which is driven by the output of a super-luminescent diode as an optical noise source to generate constant-amplitude and random-phase (CARP) light for the Drive light^38^. The CARP light is divided into two beams at a fiber coupler (FC) and each of the CARP beams is attenuated by an optical attenuator (ATT) to adjust the injection strength. The CARP beams are unidirectionally injected to Response 1 and 2 lasers though optical isolators (ISO). A tracking procedure is required to adjust for the slow variation of the timing offset between the waveform and the sampling clock.

Each of the Response 1 and 2 lasers is subject to optical feedback from a fiber mirror reflector (Ref) forming an external cavity. The external cavity lengths are set to 3.68 m (one-way) for both the Response 1 and 2 lasers, and the corresponding feedback delay time (roundtrip) is 35.4 ns. Precise matching of the external cavity lengths is required to achieve high-quality synchronization between the Response 1 and 2 lasers. The phase of the optical feedback light from each Response laser is shifted by a phase modulator (PM) with a random binary waveform, generated with an arbitrary waveform generator (AWG), with the binary values ‘0’ and ‘1’, corresponding to no and π-phase shift, respectively. Note that each Response laser has an independent phase modulator with random sequences of 0 and 1 generated independently from other chaotic semiconductor lasers^33, 34^. The output of each of the two Response lasers is detected by a photodiode (PD) (New Focus, 1554-B, 12 GHz bandwidth), amplified by an electric amplifier (Amp) (New Focus, 1422-LF, 20 GHz bandwidth), and observed by a digital oscilloscope (Tektronix, DPO71604B, 16 GHz bandwidth, 50 GigaSamples/s) and an RF spectrum analyzer (Agilent, N9010A, 26.5 GHz bandwidth).

We set the optical wavelengths of the Drive and Response lasers by adjusting the temperature of the lasers. Optical injection locking between the Drive and Response lasers is required for common-signal-induced synchronization. It is important to satisfy the following two conditions for oblivious transfer: The correlation between the intensities of the drive and response laser outputs is always low, and the correlation between the intensities of the two response lasers is high when the phase-shift parameter is the same, and low when the phase-shift parameter is different. The former condition is to prevent Bob from estimating the intensity waveform of Alice from the intensity waveform of the common driving light.

### Privacy amplification and information reconciliation for oblivious transfer

We explain a method of privacy amplification for oblivious transfer to ensure security with respect to non-ideal probabilities, noise errors or attacks. We also estimate the reduction of the key generation rate by additional procedures, such as privacy amplification and information reconciliation.

In the protocol of oblivious transfer, Alice creates two sequences ***x***_*A*,0_ and ***x***_*A,1*_, using observed bits *x*_*A*_(t) corresponding to the parameter matches *v*_*A*_(*t*) = *c*_*B*_(*t*) and *v*_*A*_(*t*) = *c*_*B*_(*t*) + 1 respectively. That is, for the sequence ***x***_*A,0*_, Alice uses the bits *x*_*A*_(*t*) observed at times *t* when *v*_*A*_(*t*) = *c*_*B*_(*t*), and for the sequence ***x***_*A,1*_, Alice uses the bits *x*_*A*_(*t*) observed at times *t* when *v*_*A*_(*t*) = *c*_*B*_(*t*) + 1. Then Alice employs the following information reconciliation^56^ and privacy amplification^48, 49^. To this end, Alice and Bob share two parity check matrices *S*_*0*_, *S*_1_ and hashing matrices *K*_0_, *K*_1_ in advance, and Alice calculates following four vectors

2 $${{\boldsymbol{s}}}_{A,0}={S}_{0}{{\boldsymbol{x}}}_{A,0}$$

3 $${{\boldsymbol{s}}}_{A,1}={S}_{1}{{\boldsymbol{x}}}_{A,1}$$

4 $${{\boldsymbol{k}}}_{A,0}={K}_{0}{{\boldsymbol{x}}}_{A,0}$$

5 $${{\boldsymbol{k}}}_{A,1}={K}_{1}{{\boldsymbol{x}}}_{A,1},$$

where ***k***_*A*,0_ and ***k***_*A*,1_ corresponds to two cryptographic keys. Alice encrypts the message *M*_0_ with the key ***k***_*A,0*_ and the message *M*_1_ with the key ***k***_*A*,1_, and sends her original parameter set {*v*_*A*_(*t*)}^*m*^_*t* =1_, two parity check vectors ***s***_*A,0*_, ***s***_*A*,1_, and the two encrypted messages to Bob. Finally, Bob obtains a vector ***x***_*B,b*_ by using the bits *x*_*B*_(*t*) for the sampling time *t* such that *v*_*B*_(*t*) = *v*_*A*_(*t*), reproduces a vector ***y***_*B,b*_ by using the relation *S* ***y***_*B,b*_ = ***s***_*b*_ and ***x***_*B,b*_, and creates a key as ***k***_*B,b*_ = *K*_*0*_ ***y***_*B,b*_, where *b* = 0 or 1 is the value Bob initially decided. Bob’s key will be identical to Alice’s key ***k***_*A,b*_ when ***x***_*A,b*_ = ***y***_*B,b*_ is satisfied, that is, errors between the bits *x*_*A*_(*t*) and *x*_*A*_(*t*) for the sampling time *t* such that *v*_*B*_(*t*) = *v*_*A*_(*t*) are corrected. Thus Bob is able to decode the message *M*_*b*_ that he intended but not the other message, and Alice does not know which of the two messages Bob can decode. It should be noted that the bias of the frequencies of 0’s and 1’s in Alice’s observed bits {*x*_*A*_(*t*)}^*m*^_*t* =1_ and the leakage of information of her bits from that of Bob’s observed bits {*x*_*B*_(*t*)}^*m*^_*t* =1_ are eliminated. Following refs 45, 57 and 58, the key generation rate *R* can be obtained as

6 $$R=(1/2)({\rm{H}}({X}_{A,b+1}|{X}_{B,b})-{\rm{H}}({X}_{A,b}|{X}_{B,b})),$$

where the factor 1/2 corresponds to the probability of the parameter match, *﻿X*_A,b_ and *X*_B,b_ denote random variables corresponding to the sample sequences **x**_A,b_ and **x**_B,b_, ﻿and H(*X* |*Y*) represents the entropy of *X* conditioned on *Y*.

### Secure comparison using Yao’s garbled circuit

We implement a multibit secure comparator with a garbled circuit. The detailed protocol of Yao’s garbled circuit is shown in the supplementary material. Alice generates the garbled circuit as follows. Alice assigns two randomly generated 100-bit strings (labels) to each wire in the circuit: one for Boolean 0 and one for 1. The random bit strings were generated using a physical random number generator, realized with a semiconductor laser^33, 34^. Next, for each gate in the circuit Alice replaces 0 and 1 in the truth tables with the corresponding 100-bit labels. She then encrypts each output of each truth table with the corresponding two input labels. The Advanced Encryption Standard (AES)^59^ block cipher is used for the encryption, and a 28-bit check string (all zeros) is added to each input and output label to test for correct decryption. Given two particular input strings at a gate, only one of the 4 output strings can be decrypted using the input labels as keys. After encrypting the table, Alice randomly permutes the table such that no one can learn the output value based on row position. Alice and Bob use oblivious transfer for each 64-bit input to obtain Bob’s garbled input.

In the case of *n*-bit secure comparison, the number of input bits is *n* for each user Alice and Bob. The number of gates and wires are given by 5*n*-5 and 7*n*-5, respectively (*n* ≥ 2), so for *n* = 64, the number of gates is 315 and the number of the wires is 443. Assigning 100-bit physical random numbers to each wire of the gates for 0 and 1 requires 88,600 random bits.

The emulation of the Yao’s garbled circuit protocol including the one-out-of-two oblivious transfer protocol is executed on a computer with a Intel(R) Core(TM) i7-4770 CPU and 8.2 GB RAM with Windows 7 Professional 64-bit operating system.

## Electronic supplementary material

Supplementary material
