# Supplementary material for: Physical implementation of oblivious transfer using optical correlated randomness
Source: Sci Rep. 2017 Aug 16;7:8444. doi: 10.1038/s41598-017-08229-x (PMC5559580; doi:10.1038/s41598-017-08229-x)
Supplement: Supplementary file 1 — Supplementary material [file 41598_2017_8229_MOESM1_ESM.pdf]

Supplementary material

Physical implementation of oblivious transfer using optical correlated randomness

Tomohiro Ito, Hayato Koizumi, Nobumitsu Suzuki, Izumi Kakesu, Kento Iwakawa, Atsushi Uchida, Takeshi Koshiba, Jun Muramatsu, Kazuyuki Yoshimura, Masanobu Inubushi, and Peter Davis

Supplementary methods

Secure computation protocol using Yao's garbled circuit

The protocol of secure computation with Yao's garbled circuit and oblivious transfer is shown in Fig. S1. We define Alice as the designer of the garbled circuit, and Bob as the evaluator of the garbled circuit.

1. First Alice generates a Boolean circuit corresponding to the desired computation. An example of a Boolean circuit for 4-bit secure comparison is shown in Fig. S2. The output of the comparator determines which input of Alice or Bob is larger.
2. Alice generates the corresponding garbled circuit. Alice assigns random labels to each entry in the Boolean truth table of each gate, replacing each 1 and 0 by a label consisting of a random bit string. An example of the assignment of random bit strings to an OR gate is shown in Fig. S3. Alice encrypts each output label of each gate using the corresponding two input labels, and shuffled the rows of the table. Alice sends the garbled circuit to Bob.
3. Alice generates her garbled input, the labels corresponding to her input bits. Alice sends her garbled input to Bob.
4. Alice and Bob use oblivious transfer for each of Bob's input bits so that Bob can obtain Bob's input labels without Alice knowing his input.
5. Bob executes the garbled circuit with Alice's and Bob's input labels, and obtains the label corresponding to the output from the final gate.
6. Alice informs Bob the labels of the outputs of the final gates so that he can decode the output.
7. Finally Bob decodes the output and sends the result to Alice.

Encryption of logic gates

Encryption is used to encrypt the output label of each logic gate using the corresponding input labels as keys. An example of encryption of a OR gate is shown in Fig. S4. $k_\alpha{}^\delta$ is the label assigned at the wire α for the bit δ (δ = 0 or 1). The input labels $k_1{}^0$ and $k_1{}^1$ are assigned for the input wire 1, the input labels of $k_2{}^0$ and $k_2{}^1$ are assigned for the input wire 2, and the output labels of $k_3{}^0$ and $k_3{}^1$ are assigned for the output wire 3. The output $k_3{}^0$ or $k_3{}^1$ is encrypted using the two labels $k_1{}^\delta$ and $k_2{}^\delta$ (δ = 0 or 1) as cryptographic keys. For example, the garbled circuit is generated as follows[1].

$$c_{i,j} = E_{k_1^i}\left(E_{k_2^j}\left(k_3^{g(i,j)}\right)\right), \tag{1}$$

where $g(i,j)$ is the output of the gate with the input bits i and j (i, j = 0 or 1). $E_k(p)$ is

the encryption of the plaintext p with the key k, and $k_3^{g(i,j)}$ is the random bit string corresponding to the output of the gate with the input bits i and j. The garbled output $k_3^{g(i,j)}$ is encrypted successively with the keys k_2^j and k_1^i. The cipher text $c_{i,j}$ indicates the garbled output corresponding to the input $w_1 = i$ and $w_2 = j$. Decryption of an output label is possible if the corresponding input labels are known, using the inverse process,

$$k_3^{g(i,j)} = D_{k_2^j}\left(D_{k_1^i}(c_{i,j})\right), \tag{2}$$

where $D_k(c)$ is the decryption of the cipher text c with the key k. The decrypted output of a gate can be used as an input label to decrypt the next gate in the logic circuit. It is important to avoid the decryption with different labels. For example, suppose Bob has the two labels k_1^0 and k_2^0, he is only able to decode $c_{0,0}$, but not others ($c_{0,1}$, $c_{1,0}$, $c_{1,1}$).

Protocol of oblivious transfer against multiple observation attack

One type of malicious-user attack on the optical correlated randomness system is the multiple observation attack[45] (also called the sampling attack[43]), where multiple optical receiving nodes can be used to identify all the possible observations of parameter-bit pairs by Bob. To avoid the multiple observation attack, the cascaded laser system has been proposed[36,37], where N lasers with phase modulators are coupled unidirectionally. The users independently shift the phase of the feedback light (0 or π) at each stage of the cascaded laser system at random, and the number of different settings of phase parameters for an N-stage cascaded laser system is 2^N. An attacker needs to use 2^N N-stage cascaded laser systems to simultaneously achieve all the possible observations. The number of cascaded laser systems required for the attack increases exponentially, and this property can be used to guarantee the information theoretic security of the system against the multiple observation attack[43,45].

The oblivious transfer protocol can be extended, as follows, to the case of multiple observation parameters. The parameter vectors $v_A = (v_{A,j})^N_{j=1}$ and $v_B = (v_{B,j})^N_{j=1}$ are N-dimensional vectors (j = 1, 2, …, N) with $v_{A,j}$ and $v_{B,j}$ having values 0 or 1 corresponding to the phase shift at each stage of the cascaded laser system. Alice and Bob decide their parameters $v_{A,j}$ and $v_{B,j}$ randomly and independently. Common random light from the drive system is injected into the first-stage laser of Alice (and Bob), and the output of j-th-stage laser for each user is injected into (j+1)-th-stage laser unidirectionally[37]. Alice and Bob extract their binary outputs x_A and x_B (0 or 1) from the

temporal intensity waveforms of the final (N-th-stage) laser outputs by sampling at a predetermined timing and quantizing their sampled values. The parameter-bit pairs (v_A, x_A) and (v_B, x_B) are stored in Alice and Bob's data recorders, respectively.

Alice creates an N-dimensional random binary vector $c_A = (c_{A,j})^N_{j=1}$ (c_A is a non-zero vector) and sends it to Bob after the observation parameter-bit pairs are generated to avoid the multiple observation attack. Bob sends the following parameter vector $c_B = (c_{B,j})^N_{j=1}$ defined as

$$c_B = v_B + b\, c_A \qquad (3)$$

to Alice if he intends to receive the message b (M_b, $b = 0$ or 1), where + denotes bitwise exclusive-OR (XOR) operation. Alice generates the key k_0 for the message M_0 from her bit x_A(t) at the sampling time t such that v_A(t) = c_B(t). Alice also generates the key k_1 for the message M_1 from her bit x_A(t) at the sampling time t such that v_A(t) = c_B(t) + c_A(t). Alice encrypts M_0 with k_0 and M_1 with k_1, respectively, and sends the two encrypted messages with her original parameter set v_A to Bob. Finally, Bob creates a key k_b using the bits x_B(t) for the sampling time t such that v_B(t) = v_A(t). Bob's key will be identical to Alice's key k_b, where $b = 0$ or 1 is the value Bob initially decided. Bob is able to decode only one of the two messages M_b, and Alice does not know which of the two messages Bob can decode. This protocol for oblivious transfer with multiple observation parameters allows the use of a multi-stage cascaded laser system that will improve security against the multiple observation attack.

We consider the situation where malicious Bob prepares a large number of the optical nodes, which are subject to a common drive signal, and each of his systems has different values of the optical feedback phase parameter[37]. To cope with the multiple observation attack, it is effective to increase the number N of the laser stages in the cascaded laser system, because Bob needs to increase exponentially the number of his cascaded laser systems to perform the perfect multiple observation attack.

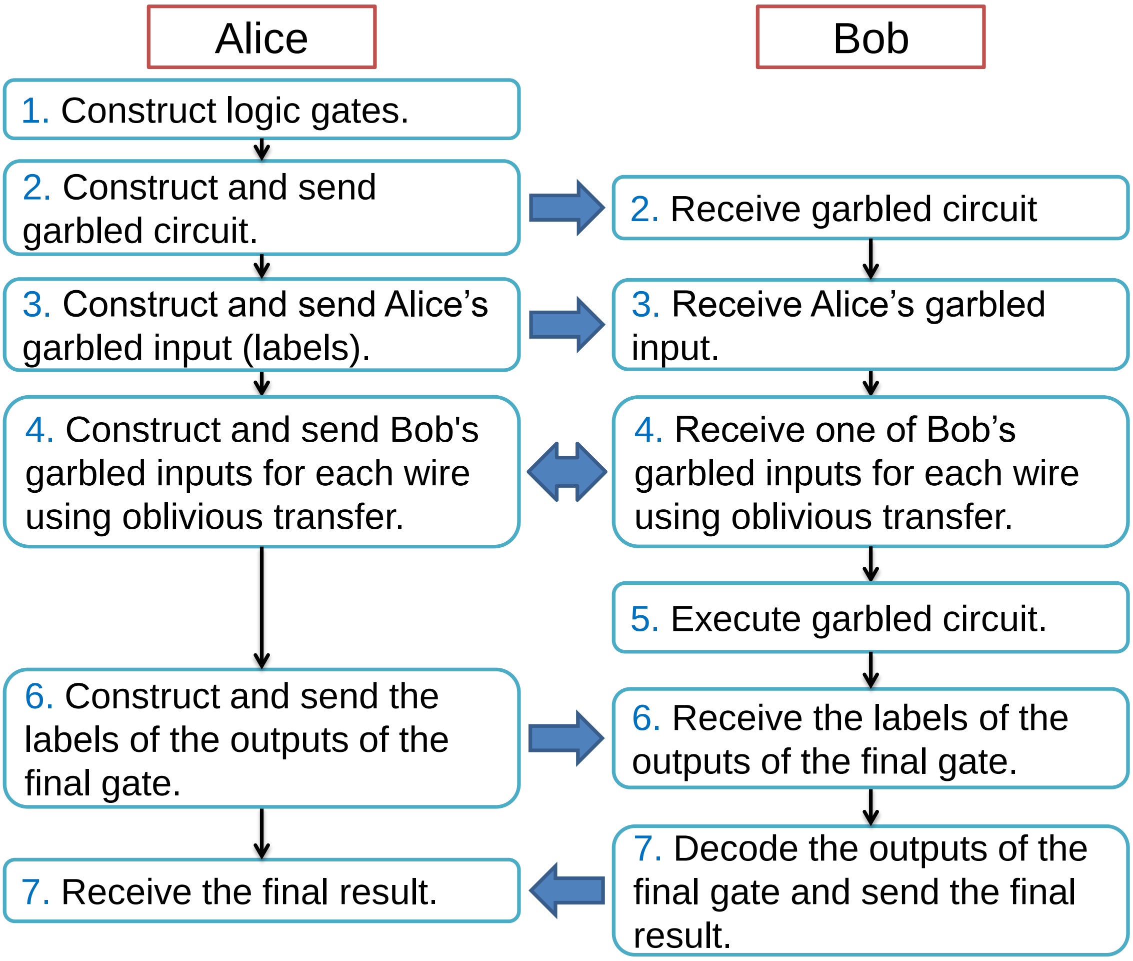

Fig. S1 Protocol of secure computation with garbled circuit and oblivious transfer.

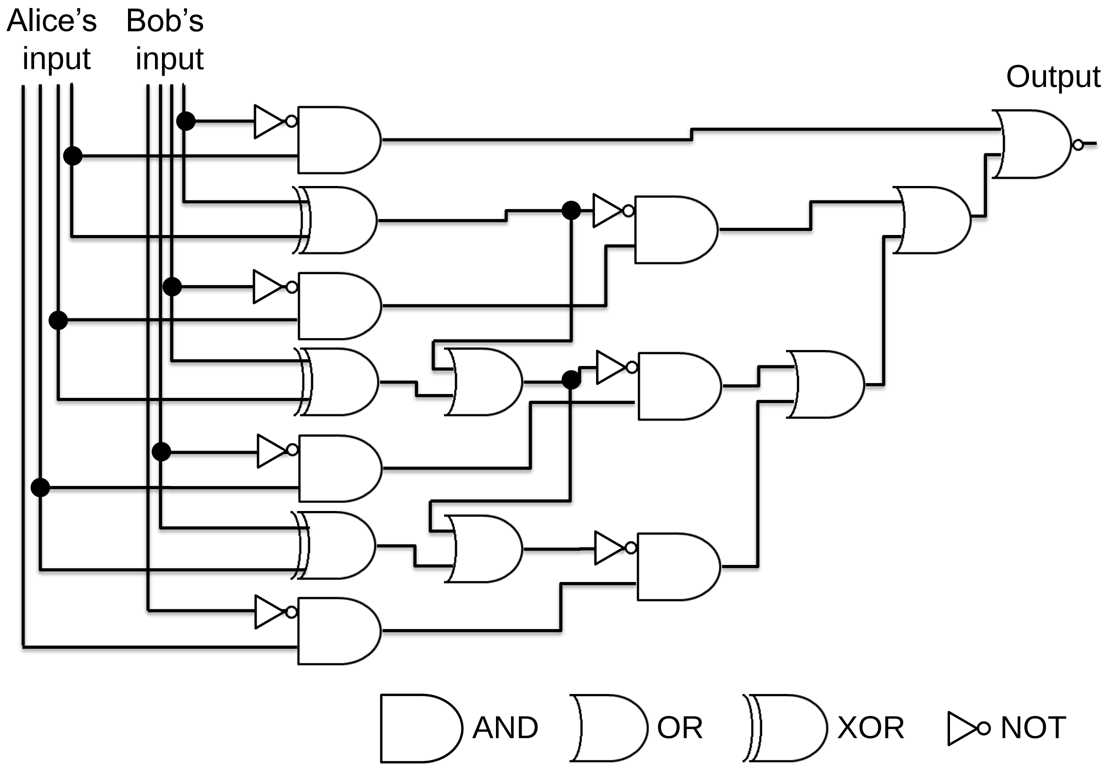

Fig. S2 Example of a 4-bit comparator with logic gates.

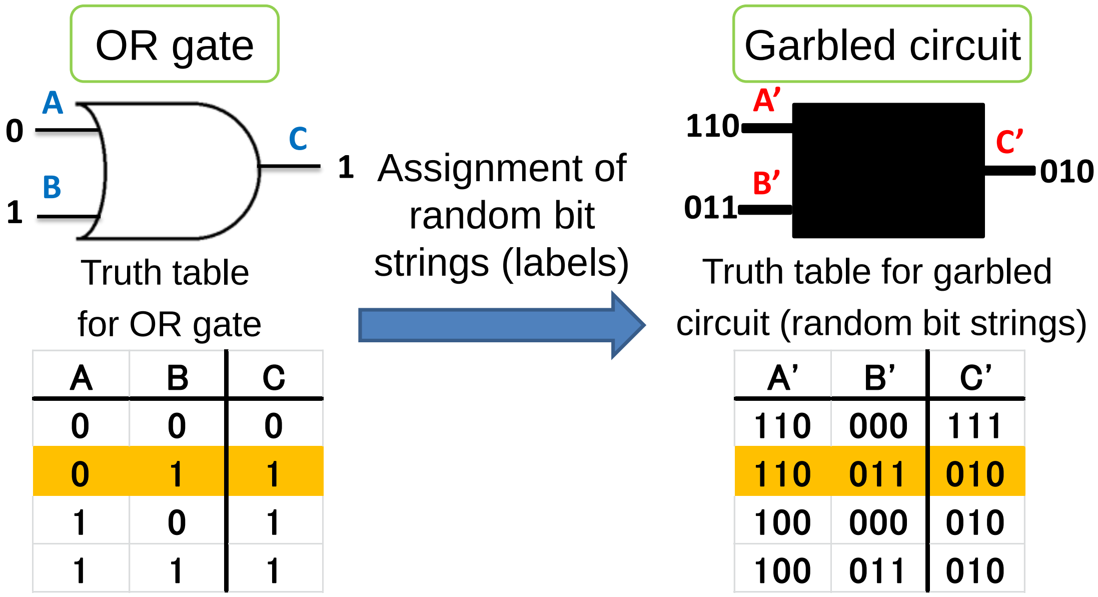

A	B	C
0	0	0
0	1	1
1	0	1
1	1	1

A’	B’	C’
110	000	111
110	011	010
100	000	010
100	011	010

Fig. S3 Example of OR gate and its garbled circuit.

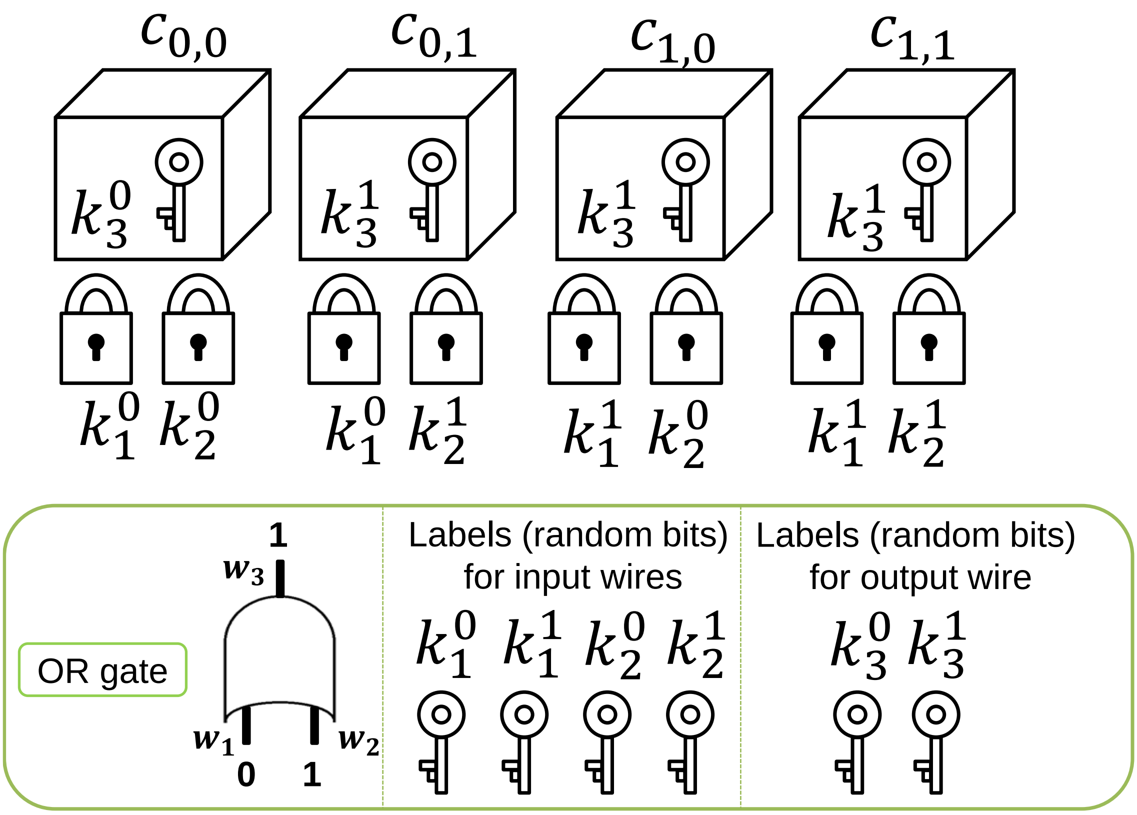

Fig. S4 Example of construction of garbled circuit (OR gate).
